# Supplementary material for: Impact of Bariatric Surgery on Subtilisin/Kexin Type 9 (PCSK9) Gene Expression and Inflammation in the Adipose Tissue of Obese Diabetic Rats
Source: Int J Mol Sci. 2023 Nov 30;24(23):16978. doi: 10.3390/ijms242316978 (PMC10707086; doi:10.3390/ijms242316978)
Supplement: Supplementary file 1 [file ijms-24-16978-s001.zip › ijms-2539534-supplementary.pdf]

Supplementary Figures

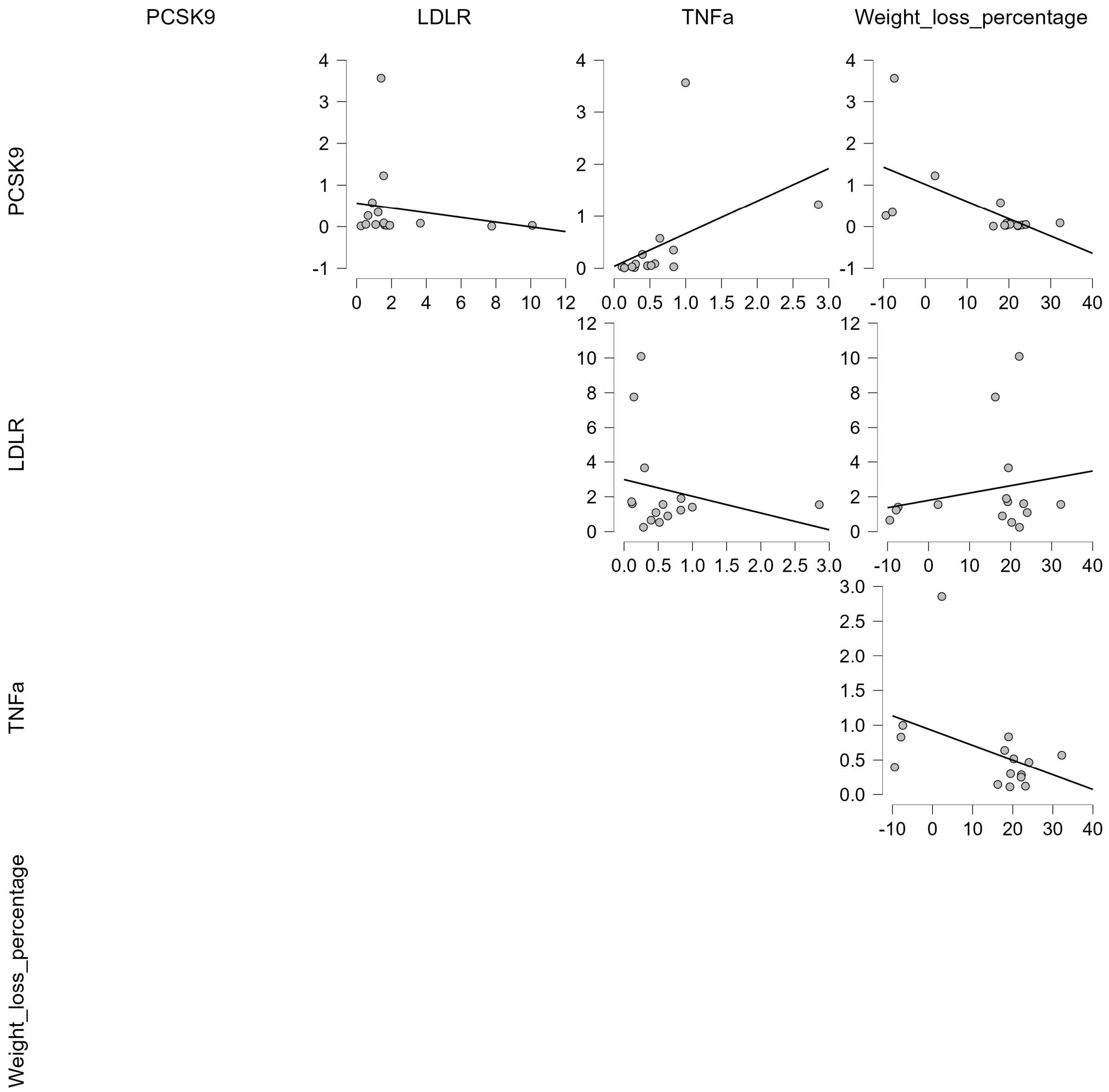

Supplementary Figure S1: Demonstrating non-significant correlations. All axes apart from weight loss percentage relate to gene expression (fold change)

Spearman's Correlations

Variable		PCSK9		TNFa	Weight_loss_percentage	LDLR
1. PCSK9	Spearman's rho	—				
	p-value	—				
2. TNFa	Spearman's rho	0.757	**	—		
	p-value	0.002		—		
3. Weight_loss_percentage	Spearman's rho	-0.464		-0.396	—	
	p-value	0.083		0.145	—	
4. LDLR	Spearman's rho	-0.368		-0.304	0.082	—
	p-value	0.178		0.271	0.773	—

* p < .05, ** p < .01, *** p < .001

Supplementary Table S1
